# {Bis[2-(pyridin-2-yl)eth­yl]amine}­dibromidocopper(II)

**DOI:** 10.1107/S2414314626006899

**Published:** 2026-07-10

**Authors:** Zachariah Bess, Celisha Oscar, Ummey Hafsa Bithi, Diego Duenas Parapar, William Jacob Lowe, Fruzsina Madzig, Yilma Gultneh, Ray J Butcher

**Affiliations:** a Department of Chemistry Howard University, 525 College St NW Washington DC 20059 USA; Benemérita Universidad Autónoma de Puebla, México

**Keywords:** crystal structure, copper structure, five coordinate structures, tridentate amine ligand

## Abstract

The crystal structure of the title complex, [CuBr_2_(C_14_H_17_N_3_)], comprises two molecules in the asymmetric unit. The five-coordinate copper(II) atoms in the two independent molecules exhibit slightly different coordination environments on the continuum between square-pyramidal and trigonal–bipyramidal, with values of τ = 0.442 and 0.574. In the extended structure, both independent mol­ecules form linked dimers with graph-set notation *R*^2^_4_(8) through N—H⋯Br and C—H⋯Br inter­actions.

## Abstract 2

Two molecules with slightly different five-coordinate Cu^II^ atoms are present in the asymmetric unit of thetitle complex.

## Graphical abstract

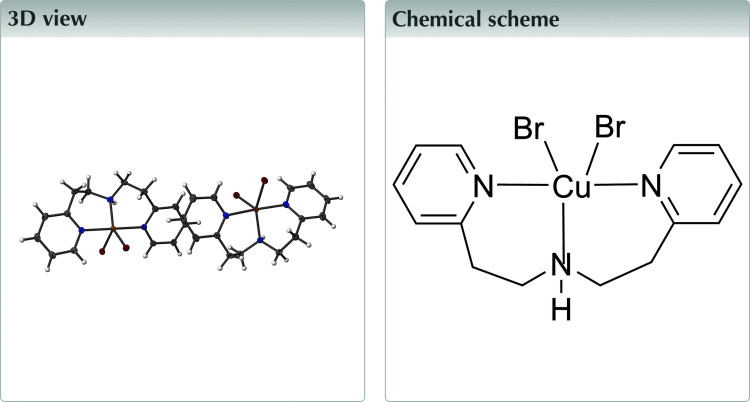

## Structure description

Ligands derived from *N*-alkyl- or *N*-aryl-bis­[2-(pyrid-2-yl)eth­yl]amines have played a central role in biomimetic coordination chemistry, particularly in delineating the mechanism of O_2_ binding and activation by copper(I) (Blackman & Tolman, 2000 ▸) and iron(II) (He *et al.*, 2007 ▸). As part of our own studies of copper coordination chemistry (Assey *et al.*, 2010*a* ▸,*b* ▸, 2011 ▸; Ayikoé *et al.*, 2011 ▸; Okeke *et al.*, 2017 ▸, 2018 ▸, 2019 ▸), we wished to use bis­[2-(pyrid-2-yl)eth­yl]amine (*L*^H^) as a precursor for some new *N*-alkyl-bis­[2-(pyrid-2-yl)eth­yl]amine complexes with copper. The title copper complex crystallizes in the monoclinic space group *P*2_1_/*n*, with two mol­ecules in the asymmetric unit (**1a** and **1b**). Even though the compound crystallizes in a centrosymmetric space group, the two amine N atoms are potentially chiral and the difference between **1a** and **1b** is the conformation about N2 in each mol­ecule, which has been inverted. Table 1 ▸ lists the bond lengths and angles associated with both mol­ecules. From this, it can be seen that these metrical parameters are very similar for both mol­ecules, with the largest deviations occurring for bond angles involving N1—Cu—N3 [175.43 (10) and 178.80 (9)° for **1a** and **1b**, respectively] and N2—Cu1—Br [148.90 (7) and 144.35 (7)° for **1a** and **1b**, respectively], Br1—Cu1—Br2 [114.68 (2) and 118.88 (2)° for **1a** and **1b**, respectively].

**Figure 1:**
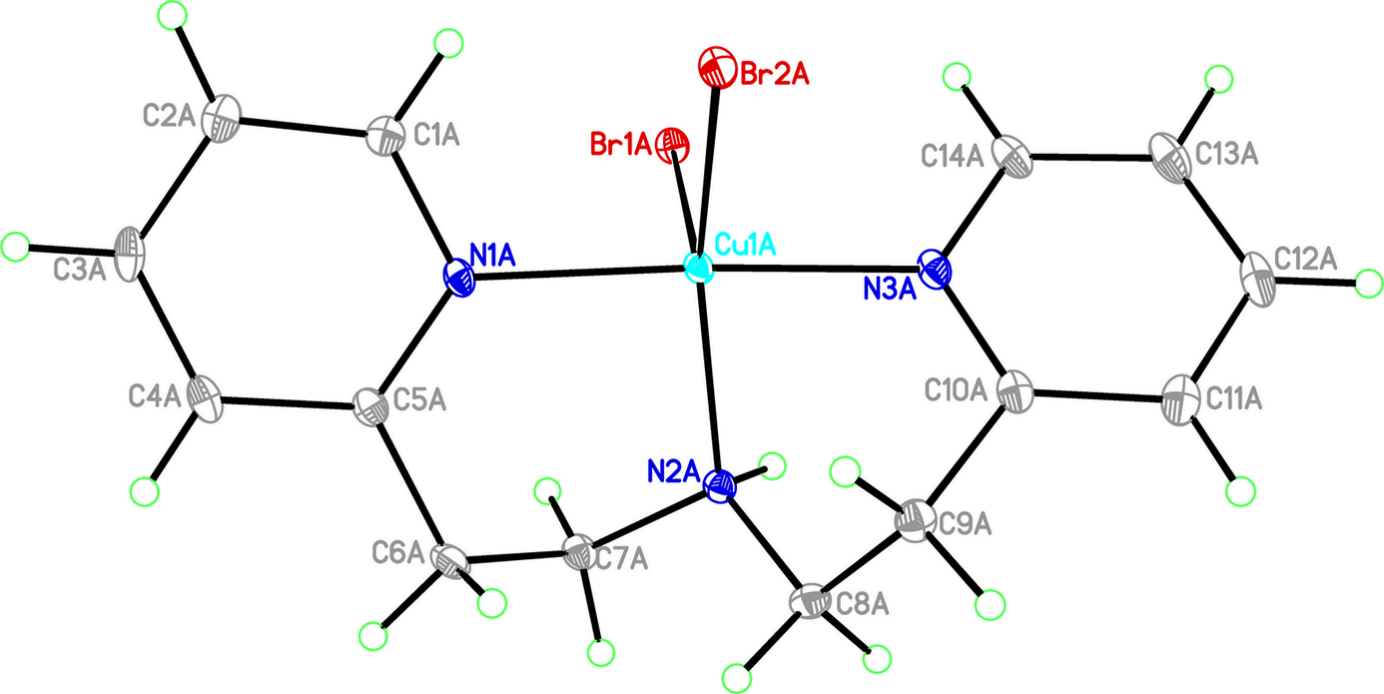
The mol­ecular structure of **1a** showing the atomic numbering scheme. Atomic displacement parameters are at the 30% probability level.

**Figure 2:**
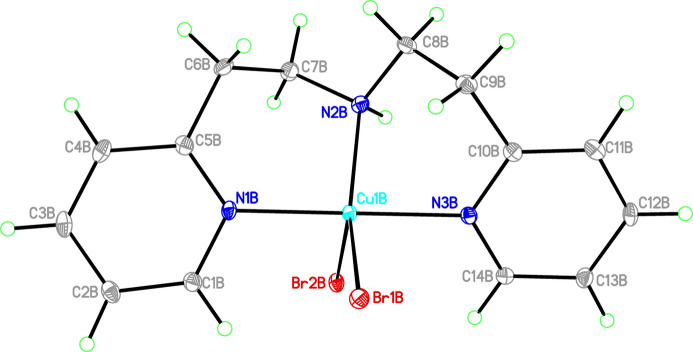
The mol­ecular structure of **1b** showing the atomic numbering scheme. Atomic displacement parameters are at the 30% probability level.

These two mol­ecules exhibit five-coordinate copper(II) atoms on the continuum between square pyramidal (τ = 0) and trigonal bipyramidal (τ = 1) (Addison *et al.*, 1984 ▸). Mol­ecule **1a** adopts a distorted square-pyramidal geometry (τ = 0.442, Fig. 1 ▸) while mol­ecule **1b** adopts a distorted trigonal–bipyramidal geometry (τ = 0.574, Fig. 2 ▸). The main difference between the two molecular structures, which results in the differing τ values, lies in the values α and β used in determining τ. All other metrical parameters are very similar. In mol­ecules **1a** and **1b**, the dihedral angles between the two pyridine rings are 34.85 (8) and 30.64 (9)°, respectively.

Similar structures have previously been reported. One is di­bromido­{bis­[2-(pyridin-2-yl)eth­yl]amine}­copper(II) (**2**), which crystallizes as a di­chloro­methane solvate (Mokuolu *et al.*, 2009 ▸). In this structure there is one shorter [2.4653 (4) Å] and one longer [2.6559 (4) Å] Cu—Br bond; these values are similar to those in the title structure. In another study (Butcher *et al.*, 2008 ▸), the structure of a similar complex was reported, di-μ-bromido-bis­({bis­[2-(2-pyrid­yl)eth­yl]amine}­copper(II)) bis­perchlorate, obtained from a reaction involving both copper perchlorate and copper bromide. Here the τ value was 0.31 and Cu—Br bond lengths show the same pattern of one being significantly shorter than the other [2.4542 (7) and 2.8908 (8) Å].

Two other similar structures are polymorphs of di­chloro­{bis­[2-(pyridin-2-yl)eth­yl]amine}­copper(II) (**3**, **4**) (Leaver *et al.*, 2003 ▸; Jopp *et al.*, 2017 ▸). Structure **3** is isotypic with **1** but has been solved in the space group *P*2_1_/*c*. This structure also contains two mol­ecules in the asymmetric unit, one of which has a τ value less than 0.5 (0.456) while the other has a τ value greater than 0.5 (0.527), which is similar to the values in the current structure. Structure **4** crystallizes in the space group *P*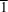 with one mol­ecule in the asymmetric unit with a τ value of 0.393. In both **3** and **4**, the Cu—Cl bond distances [2.382 (2) and 2.424 (2) Å in **3** and 2.3200 (5) and 2.4873 (5) Å in **4**] are more similar in length compared to the Cu—Br bond lengths in **1a** and **1b**.

**Figure 3:**
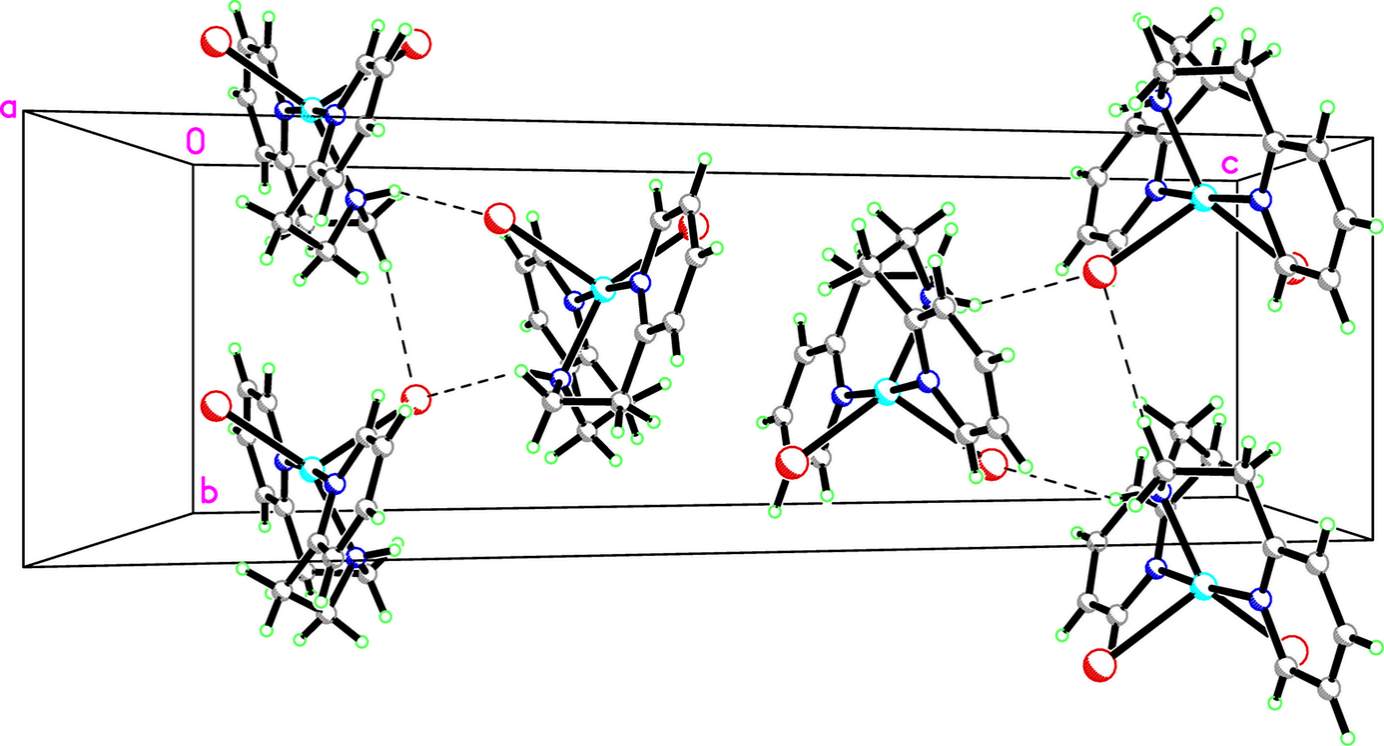
Packing diagram viewed down the *a* axis showing the hydrogen bonding as dashed lines.

Fig. 3 ▸ shows the packing of the title complex showing that both **1a** and **1b** form linked dimers with graph-set notation 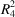
(8) (Etter *et al.*, 1990 ▸) through N—H⋯Br and C—H⋯Br inter­actions (Table 2 ▸).

## Synthesis and crystallization

The ligand, bis­[2-(pyridin-2-yl)eth­yl]amine, was synthesized using previous methods (Uhlig *et al.*, 1966 ▸; Nelson & Rodgers, 1967 ▸; Romary *et al.*, 1968 ▸; Hoorn *et al.*, 1996 ▸). The title compound was synthesized by using a methano­lic solution of copper bromide mixed with a chloro­form solution of the ligand. The resulting bright-blue solution was slowly evaporated to yield the blue crystals.

## Refinement

Crystal data, data collection and structure refinement details are summarized in Table 3 ▸.

## Supplementary Material

Crystal structure: contains datablock(s) I. DOI: 10.1107/S2414314626006899/bh4103sup1.cif

Structure factors: contains datablock(s) I. DOI: 10.1107/S2414314626006899/bh4103Isup2.hkl

CCDC reference: 2566612

Additional supporting information:  crystallographic information; 3D view; checkCIF report

## Figures and Tables

**Table 1:** Selected geometric parameters (Å, °)

Br1*A*—Cu1*A*	2.6540 (5)	Br1*B*—Cu1*B*	2.4572 (5)
Br2*A*—Cu1*A*	2.4670 (5)	Br2*B*—Cu1*B*	2.6657 (5)
Cu1*A*—N3*A*	2.017 (2)	Cu1*B*—N3*B*	2.024 (2)
Cu1*A*—N1*A*	2.046 (2)	Cu1*B*—N1*B*	2.029 (2)
Cu1*A*—N2*A*	2.053 (2)	Cu1*B*—N2*B*	2.054 (2)
			
N3*A*—Cu1*A*—N1*A*	175.43 (10)	N3*B*—Cu1*B*—N1*B*	178.80 (9)
N3*A*—Cu1*A*—N2*A*	84.36 (9)	N3*B*—Cu1*B*—N2*B*	84.69 (9)
N1*A*—Cu1*A*—N2*A*	94.20 (9)	N1*B*—Cu1*B*—N2*B*	94.72 (9)
N3*A*—Cu1*A*—Br2*A*	86.92 (7)	N3*B*—Cu1*B*—Br1*B*	88.43 (6)
N1*A*—Cu1*A*—Br2*A*	92.14 (7)	N1*B*—Cu1*B*—Br1*B*	92.65 (7)
N2*A*—Cu1*A*—Br2*A*	148.90 (7)	N2*B*—Cu1*B*—Br1*B*	144.35 (7)
N3*A*—Cu1*A*—Br1*A*	95.27 (7)	N3*B*—Cu1*B*—Br2*B*	91.90 (7)
N1*A*—Cu1*A*—Br1*A*	89.18 (6)	N1*B*—Cu1*B*—Br2*B*	87.13 (6)
N2*A*—Cu1*A*—Br1*A*	95.85 (7)	N2*B*—Cu1*B*—Br2*B*	96.32 (7)
Br2*A*—Cu1*A*—Br1*A*	114.679 (17)	Br1*B*—Cu1*B*—Br2*B*	118.880 (18)

**Table 2:** Hydrogen-bond geometry (Å, °)

*D*—H⋯*A*	*D*—H	H⋯*A*	*D*⋯*A*	*D*—H⋯*A*
N2*A*—H1*CC*⋯Br1*A*^i^	0.87 (4)	2.71 (4)	3.504 (2)	152 (3)
C1*A*—H1*A*⋯Br2*A*	0.95	2.80	3.272 (3)	112
C2*A*—H2*A*⋯Br2*A*^ii^	0.95	3.00	3.810 (3)	144
C4*A*—H4*A*⋯Br2*A*^iii^	0.95	3.09	3.849 (3)	138
C7*A*—H7*AA*⋯Br1*A*^iv^	0.99	3.05	3.985 (3)	158
C8*A*—H8*AA*⋯Br1*A*^i^	0.99	3.11	3.728 (3)	122
C14*A*—H14*A*⋯Br1*A*	0.95	2.94	3.494 (3)	118
N2*B*—H1⋯Br2*B*^v^	0.90 (4)	2.62 (4)	3.369 (2)	142 (3)
C4*B*—H4*B*⋯Br1*B*^vi^	0.95	3.01	3.730 (3)	134
C7*B*—H7*BB*⋯Br2*B*^vii^	0.99	3.09	3.988 (3)	151
C8*B*—H8*BA*⋯Br1*B*^vii^	0.99	3.02	3.937 (3)	155
C8*B*—H8*BB*⋯Br2*B*^v^	0.99	2.96	3.613 (3)	124
C9*B*—H9*BB*⋯Br2*A*^ii^	0.99	3.12	3.680 (3)	118
C13*B*—H13*B*⋯Br2*B*^viii^	0.95	3.08	3.786 (3)	132
C14*B*—H14*B*⋯Br2*B*	0.95	2.83	3.381 (3)	118

Symmetry codes: (i) 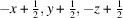; (ii) 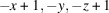; (iii) 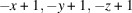; (iv) 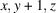; (v) 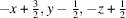; (vi) 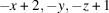; (vii) 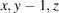; (viii) 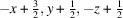.

**Table 3:** Experimental details

Crystal data	
Chemical formula	[CuBr_2_(C_14_H_17_N_3_)]
*M*_r_	450.66
Crystal system, space group	Monoclinic, *P*2_1_/*n*
Temperature (K)	100
*a*, *b*, *c* (Å)	17.88872 (12), 7.41487 (5), 24.58312 (17)
β (°)	108.4053 (7)
*V* (Å^3^)	3093.97 (4)
*Z*	8
Radiation type	Cu *K*α
μ (mm^−1^)	7.98
Crystal size (mm)	0.38 × 0.15 × 0.12

Data collection	
Diffractometer	XtaLAB Synergy, Dualflex, HyPix
Absorption correction	Multi-scan (*CrysAlis PRO*; Rigaku OD, 2024 ▸)
*T*_min_, *T*_max_	0.411, 1.000
No. of measured, independent and observed [*I* > 2σ(*I*)] reflections	93799, 6414, 6371
*R*_int_	0.124
(sin θ/λ)_max_ (Å^−1^)	0.630

Refinement	
*R*[*F*^2^ > 2σ(*F*^2^)], *wR*(*F*^2^), *S*	0.034, 0.090, 1.18
No. of reflections	6414
No. of parameters	367
H-atom treatment	H atoms treated by a mixture of independent and constrained refinement
Δρ_max_, Δρ_min_ (e Å^−3^)	0.78, −0.99

Computer programs: *CrysAlis PRO* (Rigaku OD, 2024 ▸), *SHELXT* (Sheldrick, 2015*a* ▸), *SHELXL2025/1* (Sheldrick, 2015*b* ▸) and *SHELXTL* (Sheldrick, 2008 ▸).

## full crystallographic data

### {Bis[2-(pyridin-2-yl)ethyl]amine-κ3N,N',N''}dibromidocopper(II) Crystal data

**Table:**

[CuBr_2_(C_14_H_17_N_3_)]	*F*(000) = 1768
*M**_r_* = 450.66	*D*_x_ = 1.935 Mg m^−^^3^
Monoclinic, *P*2_1_/*n*	Cu *K*α radiation, λ = 1.54184 Å
*a* = 17.88872 (12) Å	Cell parameters from 79382 reflections
*b* = 7.41487 (5) Å	θ = 2.6–76.4°
*c* = 24.58312 (17) Å	µ = 7.98 mm^−^^1^
β = 108.4053 (7)°	*T* = 100 K
*V* = 3093.97 (4) Å^3^	Needle, blue
*Z* = 8	0.38 × 0.15 × 0.12 mm

### {Bis[2-(pyridin-2-yl)ethyl]amine-κ3N,N',N''}dibromidocopper(II) Data collection

**Table:**

XtaLAB Synergy, Dualflex, HyPix diffractometer	6414 independent reflections
Radiation source: micro-focus sealed X-ray tube	6371 reflections with *I* > 2σ(*I*)
Detector resolution: 10.0000 pixels mm^-1^	*R*_int_ = 0.124
ω scans	θ_max_ = 76.4°, θ_min_ = 3.8°
Absorption correction: multi-scan (CrysAlisPro; Rigaku OD, 2024)	*h* = −22→22
*T*_min_ = 0.411, *T*_max_ = 1.000	*k* = −9→8
93799 measured reflections	*l* = −30→30

### {Bis[2-(pyridin-2-yl)ethyl]amine-κ3N,N',N''}dibromidocopper(II) Refinement

**Table:**

Refinement on *F*^2^	Primary atom site location: dual
Least-squares matrix: full	Secondary atom site location: difference Fourier map
*R*[*F*^2^ > 2σ(*F*^2^)] = 0.034	Hydrogen site location: mixed
*wR*(*F*^2^) = 0.090	H atoms treated by a mixture of independent and constrained refinement
*S* = 1.18	*w* = 1/[σ^2^(*F*_o_^2^) + (0.0398*P*)^2^ + 5.1253*P*] where *P* = (*F*_o_^2^ + 2*F*_c_^2^)/3
6414 reflections	(Δ/σ)_max_ = 0.002
367 parameters	Δρ_max_ = 0.78 e Å^−^^3^
0 restraints	Δρ_min_ = −0.99 e Å^−^^3^

### {Bis[2-(pyridin-2-yl)ethyl]amine-κ3N,N',N''}dibromidocopper(II) Special details

**Table:**

Refinement. All hydrogen atoms involved in hydrogen bonding were refined isotropically while the remaining hydrogen atoms were included in their calculated positions as a riding model. Hydrogen atoms were placed in calculated positions and refined using a riding model with isotropic displacement parameters based on those of the parent atom [C—H = 0.95 Å, *U*_iso_(H) = 1.2 *U*_eq_(C) for CH; C—H = 0.99 Å, *U*_iso_(H) = 1.2 *U*_eq_(C) for CH_2_].

### {Bis[2-(pyridin-2-yl)ethyl]amine-κ3N,N',N''}dibromidocopper(II) Fractional atomic coordinates and isotropic or equivalent isotropic displacement parameters (Å^2^)

**Table:**

	*x*	*y*	*z*	*U*_iso_*/*U*_eq_	
Br1A	0.32678 (2)	0.17070 (4)	0.29054 (2)	0.01610 (9)	
Br2A	0.29783 (2)	0.18424 (4)	0.45813 (2)	0.01666 (9)	
Cu1A	0.31782 (2)	0.36330 (5)	0.37927 (2)	0.01293 (10)	
N1A	0.43774 (13)	0.3486 (3)	0.41390 (9)	0.0142 (4)	
N2A	0.31936 (13)	0.6106 (3)	0.34208 (10)	0.0142 (4)	
H1CC	0.291 (2)	0.588 (5)	0.3068 (16)	0.021*	
N3A	0.19977 (13)	0.3931 (3)	0.34980 (9)	0.0160 (5)	
C1A	0.46619 (17)	0.1825 (4)	0.43198 (11)	0.0171 (5)	
H1A	0.429872	0.085661	0.427052	0.021*	
C2A	0.54552 (18)	0.1466 (4)	0.45729 (11)	0.0201 (6)	
H2A	0.563132	0.028590	0.470227	0.024*	
C3A	0.59871 (17)	0.2872 (4)	0.46330 (12)	0.0217 (6)	
H3A	0.653608	0.267009	0.480348	0.026*	
C4A	0.57070 (17)	0.4570 (4)	0.44413 (12)	0.0206 (6)	
H4A	0.606324	0.554664	0.447785	0.025*	
C5A	0.48950 (16)	0.4845 (4)	0.41928 (11)	0.0158 (5)	
C6A	0.45879 (17)	0.6687 (4)	0.39779 (12)	0.0196 (6)	
H6AA	0.503597	0.745049	0.396485	0.024*	
H6AB	0.435683	0.724141	0.425458	0.024*	
C7A	0.39676 (16)	0.6686 (4)	0.33848 (11)	0.0168 (5)	
H7AA	0.392124	0.791377	0.321896	0.020*	
H7AB	0.413417	0.585957	0.312848	0.020*	
C8A	0.27959 (17)	0.7578 (4)	0.36392 (12)	0.0191 (6)	
H8AA	0.248165	0.832293	0.331286	0.023*	
H8AB	0.319997	0.836380	0.389986	0.023*	
C9A	0.22570 (17)	0.6835 (4)	0.39593 (12)	0.0197 (6)	
H9AA	0.258220	0.626349	0.432049	0.024*	
H9AB	0.196262	0.784334	0.406010	0.024*	
C10A	0.16843 (16)	0.5479 (4)	0.36103 (11)	0.0166 (5)	
C11A	0.08771 (17)	0.5769 (4)	0.34179 (12)	0.0226 (6)	
H11A	0.066545	0.687629	0.349737	0.027*	
C12A	0.03832 (17)	0.4438 (5)	0.31104 (12)	0.0252 (7)	
H12A	−0.017094	0.461515	0.297484	0.030*	
C13A	0.07109 (18)	0.2841 (5)	0.30033 (13)	0.0256 (7)	
H13A	0.038273	0.189798	0.279728	0.031*	
C14A	0.15173 (18)	0.2629 (4)	0.31978 (12)	0.0214 (6)	
H14A	0.174023	0.153698	0.311813	0.026*	
Br1B	0.80932 (2)	0.36716 (4)	0.45455 (2)	0.01605 (9)	
Br2B	0.84002 (2)	0.28195 (4)	0.28522 (2)	0.01566 (9)	
Cu1B	0.80774 (2)	0.15672 (5)	0.37698 (2)	0.01267 (10)	
N1B	0.92444 (13)	0.1025 (3)	0.41115 (9)	0.0142 (4)	
N2B	0.77799 (14)	−0.0996 (3)	0.34612 (9)	0.0148 (4)	
H1	0.755 (2)	−0.077 (5)	0.3086 (16)	0.022*	
N3B	0.69146 (13)	0.2094 (3)	0.34125 (9)	0.0136 (4)	
C1B	0.97200 (17)	0.2472 (4)	0.42251 (11)	0.0177 (5)	
H1B	0.948592	0.363459	0.415936	0.021*	
C2B	1.05304 (17)	0.2359 (4)	0.44323 (12)	0.0194 (6)	
H2B	1.084565	0.341724	0.450328	0.023*	
C3B	1.08758 (16)	0.0658 (4)	0.45349 (11)	0.0208 (6)	
H3B	1.143200	0.052750	0.468059	0.025*	
C4B	1.03882 (17)	−0.0836 (4)	0.44191 (11)	0.0202 (6)	
H4B	1.061030	−0.201087	0.448752	0.024*	
C5B	0.95720 (16)	−0.0630 (4)	0.42025 (11)	0.0161 (5)	
C6B	0.90523 (18)	−0.2261 (4)	0.40605 (12)	0.0205 (6)	
H6BA	0.877525	−0.238661	0.434918	0.025*	
H6BB	0.938530	−0.334401	0.408658	0.025*	
C7B	0.84444 (17)	−0.2190 (4)	0.34666 (12)	0.0187 (6)	
H7BA	0.869628	−0.174055	0.318748	0.022*	
H7BB	0.824356	−0.342071	0.334852	0.022*	
C8B	0.72085 (17)	−0.1916 (4)	0.36938 (12)	0.0189 (6)	
H8BA	0.749723	−0.273168	0.400905	0.023*	
H8BB	0.684633	−0.266046	0.338864	0.023*	
C9B	0.67279 (17)	−0.0570 (4)	0.39186 (11)	0.0182 (5)	
H9BA	0.629352	−0.122258	0.400170	0.022*	
H9BB	0.707035	−0.005268	0.428358	0.022*	
C10B	0.63851 (16)	0.0945 (4)	0.35088 (11)	0.0157 (5)	
C11B	0.55831 (17)	0.1209 (4)	0.32579 (12)	0.0185 (6)	
H11B	0.521860	0.036644	0.332020	0.022*	
C12B	0.53139 (16)	0.2713 (4)	0.29146 (12)	0.0183 (6)	
H12B	0.476525	0.291534	0.274228	0.022*	
C13B	0.58594 (17)	0.3914 (4)	0.28277 (12)	0.0187 (6)	
H13B	0.569234	0.496742	0.260215	0.022*	
C14B	0.66554 (17)	0.3543 (4)	0.30777 (12)	0.0166 (5)	
H14B	0.703077	0.434323	0.300945	0.020*	

### {Bis[2-(pyridin-2-yl)ethyl]amine-κ3N,N',N''}dibromidocopper(II) Atomic displacement parameters (Å^2^)

**Table:**

	*U*^11^	*U*^22^	*U*^33^	*U*^12^	*U*^13^	*U*^23^
Br1A	0.01602 (15)	0.01823 (15)	0.01258 (14)	−0.00133 (10)	0.00242 (11)	−0.00240 (10)
Br2A	0.01530 (15)	0.02096 (16)	0.01303 (14)	−0.00162 (10)	0.00349 (11)	0.00407 (10)
Cu1A	0.01088 (19)	0.0141 (2)	0.01186 (19)	−0.00180 (14)	0.00080 (15)	0.00170 (14)
N1A	0.0120 (10)	0.0186 (11)	0.0103 (10)	−0.0026 (9)	0.0010 (8)	−0.0003 (8)
N2A	0.0140 (11)	0.0146 (11)	0.0128 (10)	−0.0009 (8)	0.0028 (8)	0.0004 (8)
N3A	0.0130 (11)	0.0208 (12)	0.0117 (10)	−0.0023 (9)	0.0003 (8)	0.0026 (9)
C1A	0.0187 (14)	0.0199 (13)	0.0137 (12)	0.0005 (11)	0.0065 (10)	0.0019 (10)
C2A	0.0204 (14)	0.0275 (15)	0.0122 (12)	0.0036 (11)	0.0051 (10)	0.0015 (11)
C3A	0.0129 (13)	0.0358 (17)	0.0136 (12)	0.0027 (12)	0.0000 (10)	−0.0021 (11)
C4A	0.0151 (13)	0.0283 (15)	0.0169 (12)	−0.0051 (11)	0.0029 (10)	−0.0042 (11)
C5A	0.0157 (13)	0.0183 (13)	0.0125 (11)	−0.0021 (10)	0.0034 (10)	−0.0023 (10)
C6A	0.0192 (14)	0.0165 (13)	0.0215 (14)	−0.0065 (11)	0.0042 (11)	−0.0017 (11)
C7A	0.0164 (13)	0.0186 (13)	0.0165 (13)	−0.0021 (10)	0.0068 (10)	0.0021 (10)
C8A	0.0216 (14)	0.0148 (13)	0.0211 (13)	0.0005 (11)	0.0072 (11)	−0.0026 (11)
C9A	0.0201 (14)	0.0209 (14)	0.0191 (13)	0.0005 (11)	0.0075 (11)	−0.0019 (11)
C10A	0.0154 (12)	0.0231 (14)	0.0117 (11)	−0.0012 (11)	0.0051 (9)	0.0038 (10)
C11A	0.0191 (14)	0.0314 (16)	0.0182 (13)	0.0037 (12)	0.0074 (11)	0.0065 (12)
C12A	0.0133 (13)	0.0412 (18)	0.0204 (13)	−0.0032 (12)	0.0043 (11)	0.0081 (13)
C13A	0.0195 (15)	0.0364 (18)	0.0187 (14)	−0.0105 (13)	0.0027 (11)	−0.0016 (12)
C14A	0.0194 (14)	0.0260 (15)	0.0178 (13)	−0.0087 (12)	0.0046 (11)	−0.0019 (11)
Br1B	0.01639 (15)	0.01632 (15)	0.01312 (14)	0.00041 (10)	0.00135 (11)	−0.00212 (10)
Br2B	0.01310 (14)	0.02055 (16)	0.01192 (14)	0.00031 (10)	0.00195 (10)	0.00281 (10)
Cu1B	0.0107 (2)	0.0127 (2)	0.01236 (19)	−0.00003 (14)	0.00032 (15)	−0.00011 (14)
N1B	0.0116 (10)	0.0173 (11)	0.0115 (10)	0.0015 (8)	0.0006 (8)	0.0017 (8)
N2B	0.0165 (11)	0.0143 (11)	0.0118 (10)	0.0006 (9)	0.0018 (8)	−0.0001 (8)
N3B	0.0127 (10)	0.0146 (10)	0.0117 (10)	−0.0011 (8)	0.0012 (8)	−0.0010 (8)
C1B	0.0176 (13)	0.0186 (13)	0.0140 (12)	−0.0013 (11)	0.0010 (10)	0.0018 (10)
C2B	0.0172 (14)	0.0253 (15)	0.0140 (12)	−0.0041 (11)	0.0025 (10)	0.0012 (11)
C3B	0.0136 (12)	0.0337 (16)	0.0136 (12)	0.0019 (12)	0.0023 (10)	0.0013 (11)
C4B	0.0213 (14)	0.0228 (14)	0.0148 (12)	0.0074 (11)	0.0033 (10)	0.0015 (11)
C5B	0.0179 (13)	0.0190 (13)	0.0096 (11)	−0.0017 (11)	0.0017 (9)	−0.0009 (10)
C6B	0.0218 (14)	0.0128 (13)	0.0218 (14)	0.0017 (11)	−0.0003 (11)	0.0027 (10)
C7B	0.0187 (13)	0.0172 (13)	0.0180 (13)	0.0009 (11)	0.0026 (11)	−0.0037 (10)
C8B	0.0175 (13)	0.0168 (13)	0.0212 (13)	−0.0036 (11)	0.0044 (11)	0.0031 (11)
C9B	0.0203 (13)	0.0189 (13)	0.0155 (12)	−0.0023 (11)	0.0058 (10)	0.0041 (11)
C10B	0.0168 (13)	0.0170 (13)	0.0129 (11)	−0.0017 (10)	0.0043 (10)	−0.0025 (10)
C11B	0.0195 (14)	0.0220 (14)	0.0154 (12)	−0.0053 (11)	0.0076 (10)	−0.0034 (11)
C12B	0.0111 (12)	0.0262 (15)	0.0154 (12)	0.0020 (11)	0.0008 (10)	−0.0020 (11)
C13B	0.0156 (13)	0.0204 (14)	0.0191 (13)	0.0028 (11)	0.0038 (10)	0.0032 (11)
C14B	0.0164 (13)	0.0146 (13)	0.0185 (13)	−0.0005 (10)	0.0049 (10)	0.0007 (10)

### {Bis[2-(pyridin-2-yl)ethyl]amine-κ3N,N',N''}dibromidocopper(II) Geometric parameters (Å, º)

**Table:**

Br1A—Cu1A	2.6540 (5)	Br1B—Cu1B	2.4572 (5)
Br2A—Cu1A	2.4670 (5)	Br2B—Cu1B	2.6657 (5)
Cu1A—N3A	2.017 (2)	Cu1B—N3B	2.024 (2)
Cu1A—N1A	2.046 (2)	Cu1B—N1B	2.029 (2)
Cu1A—N2A	2.053 (2)	Cu1B—N2B	2.054 (2)
N1A—C5A	1.347 (4)	N1B—C1B	1.342 (4)
N1A—C1A	1.353 (4)	N1B—C5B	1.348 (4)
N2A—C7A	1.479 (3)	N2B—C7B	1.479 (4)
N2A—C8A	1.492 (4)	N2B—C8B	1.485 (4)
N2A—H1CC	0.87 (4)	N2B—H1	0.90 (4)
N3A—C10A	1.344 (4)	N3B—C14B	1.344 (3)
N3A—C14A	1.347 (4)	N3B—C10B	1.350 (4)
C1A—C2A	1.384 (4)	C1B—C2B	1.379 (4)
C1A—H1A	0.9500	C1B—H1B	0.9500
C2A—C3A	1.388 (4)	C2B—C3B	1.392 (4)
C2A—H2A	0.9500	C2B—H2B	0.9500
C3A—C4A	1.382 (4)	C3B—C4B	1.383 (4)
C3A—H3A	0.9500	C3B—H3B	0.9500
C4A—C5A	1.402 (4)	C4B—C5B	1.396 (4)
C4A—H4A	0.9500	C4B—H4B	0.9500
C5A—C6A	1.504 (4)	C5B—C6B	1.498 (4)
C6A—C7A	1.528 (4)	C6B—C7B	1.521 (4)
C6A—H6AA	0.9900	C6B—H6BA	0.9900
C6A—H6AB	0.9900	C6B—H6BB	0.9900
C7A—H7AA	0.9900	C7B—H7BA	0.9900
C7A—H7AB	0.9900	C7B—H7BB	0.9900
C8A—C9A	1.527 (4)	C8B—C9B	1.530 (4)
C8A—H8AA	0.9900	C8B—H8BA	0.9900
C8A—H8AB	0.9900	C8B—H8BB	0.9900
C9A—C10A	1.497 (4)	C9B—C10B	1.504 (4)
C9A—H9AA	0.9900	C9B—H9BA	0.9900
C9A—H9AB	0.9900	C9B—H9BB	0.9900
C10A—C11A	1.387 (4)	C10B—C11B	1.385 (4)
C11A—C12A	1.380 (4)	C11B—C12B	1.390 (4)
C11A—H11A	0.9500	C11B—H11B	0.9500
C12A—C13A	1.383 (5)	C12B—C13B	1.386 (4)
C12A—H12A	0.9500	C12B—H12B	0.9500
C13A—C14A	1.378 (4)	C13B—C14B	1.389 (4)
C13A—H13A	0.9500	C13B—H13B	0.9500
C14A—H14A	0.9500	C14B—H14B	0.9500
			
N3A—Cu1A—N1A	175.43 (10)	N3B—Cu1B—N1B	178.80 (9)
N3A—Cu1A—N2A	84.36 (9)	N3B—Cu1B—N2B	84.69 (9)
N1A—Cu1A—N2A	94.20 (9)	N1B—Cu1B—N2B	94.72 (9)
N3A—Cu1A—Br2A	86.92 (7)	N3B—Cu1B—Br1B	88.43 (6)
N1A—Cu1A—Br2A	92.14 (7)	N1B—Cu1B—Br1B	92.65 (7)
N2A—Cu1A—Br2A	148.90 (7)	N2B—Cu1B—Br1B	144.35 (7)
N3A—Cu1A—Br1A	95.27 (7)	N3B—Cu1B—Br2B	91.90 (7)
N1A—Cu1A—Br1A	89.18 (6)	N1B—Cu1B—Br2B	87.13 (6)
N2A—Cu1A—Br1A	95.85 (7)	N2B—Cu1B—Br2B	96.32 (7)
Br2A—Cu1A—Br1A	114.679 (17)	Br1B—Cu1B—Br2B	118.880 (18)
C5A—N1A—C1A	118.3 (2)	C1B—N1B—C5B	118.6 (2)
C5A—N1A—Cu1A	126.79 (19)	C1B—N1B—Cu1B	115.44 (19)
C1A—N1A—Cu1A	114.93 (18)	C5B—N1B—Cu1B	125.84 (18)
C7A—N2A—C8A	111.8 (2)	C7B—N2B—C8B	111.8 (2)
C7A—N2A—Cu1A	115.52 (17)	C7B—N2B—Cu1B	115.90 (17)
C8A—N2A—Cu1A	114.70 (17)	C8B—N2B—Cu1B	114.36 (18)
C7A—N2A—H1CC	105 (2)	C7B—N2B—H1	103 (2)
C8A—N2A—H1CC	108 (2)	C8B—N2B—H1	110 (2)
Cu1A—N2A—H1CC	101 (2)	Cu1B—N2B—H1	101 (2)
C10A—N3A—C14A	119.3 (2)	C14B—N3B—C10B	119.1 (2)
C10A—N3A—Cu1A	118.52 (18)	C14B—N3B—Cu1B	121.92 (19)
C14A—N3A—Cu1A	122.2 (2)	C10B—N3B—Cu1B	118.95 (18)
N1A—C1A—C2A	123.3 (3)	N1B—C1B—C2B	123.5 (3)
N1A—C1A—H1A	118.4	N1B—C1B—H1B	118.3
C2A—C1A—H1A	118.4	C2B—C1B—H1B	118.3
C1A—C2A—C3A	118.3 (3)	C1B—C2B—C3B	118.5 (3)
C1A—C2A—H2A	120.8	C1B—C2B—H2B	120.8
C3A—C2A—H2A	120.8	C3B—C2B—H2B	120.8
C4A—C3A—C2A	119.1 (3)	C4B—C3B—C2B	118.3 (3)
C4A—C3A—H3A	120.5	C4B—C3B—H3B	120.9
C2A—C3A—H3A	120.5	C2B—C3B—H3B	120.9
C3A—C4A—C5A	119.7 (3)	C3B—C4B—C5B	120.4 (3)
C3A—C4A—H4A	120.1	C3B—C4B—H4B	119.8
C5A—C4A—H4A	120.1	C5B—C4B—H4B	119.8
N1A—C5A—C4A	121.3 (3)	N1B—C5B—C4B	120.7 (3)
N1A—C5A—C6A	118.8 (2)	N1B—C5B—C6B	119.4 (2)
C4A—C5A—C6A	119.9 (3)	C4B—C5B—C6B	119.9 (3)
C5A—C6A—C7A	114.2 (2)	C5B—C6B—C7B	113.5 (2)
C5A—C6A—H6AA	108.7	C5B—C6B—H6BA	108.9
C7A—C6A—H6AA	108.7	C7B—C6B—H6BA	108.9
C5A—C6A—H6AB	108.7	C5B—C6B—H6BB	108.9
C7A—C6A—H6AB	108.7	C7B—C6B—H6BB	108.9
H6AA—C6A—H6AB	107.6	H6BA—C6B—H6BB	107.7
N2A—C7A—C6A	110.8 (2)	N2B—C7B—C6B	111.0 (2)
N2A—C7A—H7AA	109.5	N2B—C7B—H7BA	109.4
C6A—C7A—H7AA	109.5	C6B—C7B—H7BA	109.4
N2A—C7A—H7AB	109.5	N2B—C7B—H7BB	109.4
C6A—C7A—H7AB	109.5	C6B—C7B—H7BB	109.4
H7AA—C7A—H7AB	108.1	H7BA—C7B—H7BB	108.0
N2A—C8A—C9A	111.8 (2)	N2B—C8B—C9B	111.9 (2)
N2A—C8A—H8AA	109.2	N2B—C8B—H8BA	109.2
C9A—C8A—H8AA	109.2	C9B—C8B—H8BA	109.2
N2A—C8A—H8AB	109.2	N2B—C8B—H8BB	109.2
C9A—C8A—H8AB	109.2	C9B—C8B—H8BB	109.2
H8AA—C8A—H8AB	107.9	H8BA—C8B—H8BB	107.9
C10A—C9A—C8A	112.1 (2)	C10B—C9B—C8B	113.7 (2)
C10A—C9A—H9AA	109.2	C10B—C9B—H9BA	108.8
C8A—C9A—H9AA	109.2	C8B—C9B—H9BA	108.8
C10A—C9A—H9AB	109.2	C10B—C9B—H9BB	108.8
C8A—C9A—H9AB	109.2	C8B—C9B—H9BB	108.8
H9AA—C9A—H9AB	107.9	H9BA—C9B—H9BB	107.7
N3A—C10A—C11A	121.3 (3)	N3B—C10B—C11B	121.2 (3)
N3A—C10A—C9A	115.9 (2)	N3B—C10B—C9B	115.4 (2)
C11A—C10A—C9A	122.8 (3)	C11B—C10B—C9B	123.4 (3)
C12A—C11A—C10A	119.6 (3)	C10B—C11B—C12B	119.7 (3)
C12A—C11A—H11A	120.2	C10B—C11B—H11B	120.1
C10A—C11A—H11A	120.2	C12B—C11B—H11B	120.1
C11A—C12A—C13A	118.7 (3)	C13B—C12B—C11B	118.9 (3)
C11A—C12A—H12A	120.7	C13B—C12B—H12B	120.6
C13A—C12A—H12A	120.7	C11B—C12B—H12B	120.6
C14A—C13A—C12A	119.5 (3)	C12B—C13B—C14B	118.6 (3)
C14A—C13A—H13A	120.3	C12B—C13B—H13B	120.7
C12A—C13A—H13A	120.3	C14B—C13B—H13B	120.7
N3A—C14A—C13A	121.7 (3)	N3B—C14B—C13B	122.5 (3)
N3A—C14A—H14A	119.2	N3B—C14B—H14B	118.8
C13A—C14A—H14A	119.2	C13B—C14B—H14B	118.8
			
C5A—N1A—C1A—C2A	2.0 (4)	C5B—N1B—C1B—C2B	−0.1 (4)
Cu1A—N1A—C1A—C2A	−179.0 (2)	Cu1B—N1B—C1B—C2B	−176.8 (2)
N1A—C1A—C2A—C3A	−1.4 (4)	N1B—C1B—C2B—C3B	−0.7 (4)
C1A—C2A—C3A—C4A	0.3 (4)	C1B—C2B—C3B—C4B	0.5 (4)
C2A—C3A—C4A—C5A	0.2 (4)	C2B—C3B—C4B—C5B	0.3 (4)
C1A—N1A—C5A—C4A	−1.5 (4)	C1B—N1B—C5B—C4B	1.0 (4)
Cu1A—N1A—C5A—C4A	179.75 (19)	Cu1B—N1B—C5B—C4B	177.32 (19)
C1A—N1A—C5A—C6A	178.2 (2)	C1B—N1B—C5B—C6B	−178.0 (3)
Cu1A—N1A—C5A—C6A	−0.5 (4)	Cu1B—N1B—C5B—C6B	−1.7 (4)
C3A—C4A—C5A—N1A	0.4 (4)	C3B—C4B—C5B—N1B	−1.1 (4)
C3A—C4A—C5A—C6A	−179.3 (3)	C3B—C4B—C5B—C6B	177.9 (3)
N1A—C5A—C6A—C7A	−45.5 (4)	N1B—C5B—C6B—C7B	48.6 (4)
C4A—C5A—C6A—C7A	134.3 (3)	C4B—C5B—C6B—C7B	−130.5 (3)
C8A—N2A—C7A—C6A	77.4 (3)	C8B—N2B—C7B—C6B	−80.2 (3)
Cu1A—N2A—C7A—C6A	−56.2 (3)	Cu1B—N2B—C7B—C6B	53.3 (3)
C5A—C6A—C7A—N2A	77.5 (3)	C5B—C6B—C7B—N2B	−77.5 (3)
C7A—N2A—C8A—C9A	−150.9 (2)	C7B—N2B—C8B—C9B	156.6 (2)
Cu1A—N2A—C8A—C9A	−16.9 (3)	Cu1B—N2B—C8B—C9B	22.4 (3)
N2A—C8A—C9A—C10A	−52.9 (3)	N2B—C8B—C9B—C10B	48.2 (3)
C14A—N3A—C10A—C11A	−0.7 (4)	C14B—N3B—C10B—C11B	1.6 (4)
Cu1A—N3A—C10A—C11A	179.8 (2)	Cu1B—N3B—C10B—C11B	−177.7 (2)
C14A—N3A—C10A—C9A	178.4 (2)	C14B—N3B—C10B—C9B	−176.2 (2)
Cu1A—N3A—C10A—C9A	−1.1 (3)	Cu1B—N3B—C10B—C9B	4.5 (3)
C8A—C9A—C10A—N3A	66.3 (3)	C8B—C9B—C10B—N3B	−66.6 (3)
C8A—C9A—C10A—C11A	−114.5 (3)	C8B—C9B—C10B—C11B	115.7 (3)
N3A—C10A—C11A—C12A	0.7 (4)	N3B—C10B—C11B—C12B	−2.0 (4)
C9A—C10A—C11A—C12A	−178.4 (3)	C9B—C10B—C11B—C12B	175.6 (3)
C10A—C11A—C12A—C13A	0.1 (4)	C10B—C11B—C12B—C13B	0.5 (4)
C11A—C12A—C13A—C14A	−0.9 (4)	C11B—C12B—C13B—C14B	1.4 (4)
C10A—N3A—C14A—C13A	−0.1 (4)	C10B—N3B—C14B—C13B	0.4 (4)
Cu1A—N3A—C14A—C13A	179.4 (2)	Cu1B—N3B—C14B—C13B	179.7 (2)
C12A—C13A—C14A—N3A	0.9 (5)	C12B—C13B—C14B—N3B	−1.9 (4)

### {Bis[2-(pyridin-2-yl)ethyl]amine-κ3N,N',N''}dibromidocopper(II) Hydrogen-bond geometry (Å, º)

**Table:**

*D*—H···*A*	*D*—H	H···*A*	*D*···*A*	*D*—H···*A*
N2*A*—H1*CC*···Br1*A*^i^	0.87 (4)	2.71 (4)	3.504 (2)	152 (3)
C1*A*—H1*A*···Br2*A*	0.95	2.80	3.272 (3)	112
C2*A*—H2*A*···Br2*A*^ii^	0.95	3.00	3.810 (3)	144
C4*A*—H4*A*···Br2*A*^iii^	0.95	3.09	3.849 (3)	138
C7*A*—H7*AA*···Br1*A*^iv^	0.99	3.05	3.985 (3)	158
C8*A*—H8*AA*···Br1*A*^i^	0.99	3.11	3.728 (3)	122
C14*A*—H14*A*···Br1*A*	0.95	2.94	3.494 (3)	118
N2*B*—H1···Br2*B*^v^	0.90 (4)	2.62 (4)	3.369 (2)	142 (3)
C4*B*—H4*B*···Br1*B*^vi^	0.95	3.01	3.730 (3)	134
C7*B*—H7*BB*···Br2*B*^vii^	0.99	3.09	3.988 (3)	151
C8*B*—H8*BA*···Br1*B*^vii^	0.99	3.02	3.937 (3)	155
C8*B*—H8*BB*···Br2*B*^v^	0.99	2.96	3.613 (3)	124
C9*B*—H9*BB*···Br2*A*^ii^	0.99	3.12	3.680 (3)	118
C13*B*—H13*B*···Br2*B*^viii^	0.95	3.08	3.786 (3)	132
C14*B*—H14*B*···Br2*B*	0.95	2.83	3.381 (3)	118

Symmetry codes: (i) −*x*+1/2, *y*+1/2, −*z*+1/2; (ii) −*x*+1, −*y*, −*z*+1; (iii) −*x*+1, −*y*+1, −*z*+1; (iv) *x*, *y*+1, *z*; (v) −*x*+3/2, *y*−1/2, −*z*+1/2; (vi) −*x*+2, −*y*, −*z*+1; (vii) *x*, *y*−1, *z*; (viii) −*x*+3/2, *y*+1/2, −*z*+1/2.

## References

[bb1] Addison, A. W., Rao, T. N., Reedijk, J., van Rijn, J. & Verschoor, G. C. (1984). *J. Chem. Soc. Dalton Trans.* pp. 1349–1356.

[bb2] Assey, G. E., Butcher, A. M., Butcher, R. J. & Gultneh, Y. (2010*b*). *Acta Cryst.* E**66**, m1475. 10.1107/S1600536810042923 PMC3009097 21588891

[bb3] Assey, G., Butcher, R. J. & Gultneh, Y. (2010*a*). *Acta Cryst.* E**66**, m653. 10.1107/S1600536810017137 PMC2979637 21579299

[bb4] Assey, G., Butcher, R. J. & Gultneh, Y. (2011). *Acta Cryst.* E**67**, m1197–m1198. 10.1107/S160053681103090X PMC3200975 22058844

[bb5] Ayikoé, K., Gultneh, Y. & Butcher, R. J. (2011). *Acta Cryst.* E**67**, m1211.

[bb6] Blackman, A. G. & Tolman, W. B. (2000). *Struct. Bonding (Berlin)* **97**, 179–211.

[bb7] Butcher, R. J., Gultneh, Y., Yisgedu, T. B. & Tesema, Y. T. (2008). *Acta Cryst.* E**64**, m323. 10.1107/S1600536807068663 PMC2960247 21201292

[bb8] Etter, M. C., MacDonald, J. C. & Bernstein, J. (1990). *Acta Cryst.* B**46**, 256–262. 10.1107/s0108768189012929 2344397

[bb9] He, C., Barrios, A. M., Lee, D., Kuzelka, J., Davydov, R. M. & Lippard, S. J. (2000). *J. Am. Chem. Soc.* **122**, 12683–12690.

[bb10] Hoorn, H. J., de Joode, P., Driessen, W. L. & Reedijk, J. (1996). *Recl Trav. Chim. Pays Bas* **115**, 191–197.

[bb11] Jopp, M., Becker, J., Becker, S., Miska, A., Gandin, V., Marzano, C. & Schindler, S. (2017). *Eur. J. Med. Chem.* **132**, 274–281. 10.1016/j.ejmech.2017.03.019 28371639

[bb12] Leaver, S. A., Palaniandavar, M., Kilner, C. A. & Halcrow, M. A. (2003). *Dalton Trans.* pp. 4224–4225.

[bb13] Mokuolu, Q. F., Kilner, C. A., McGowan, P. C. & Halcrow, M. A. (2009). *CSD Communication (*refcode OJULIP). CCDC, Cambridge, England.

[bb14] Nelson, S. M. & Rodgers, J. (1967). *Inorg. Chem.* **6**, 1390–1395.

[bb15] Okeke, U., Gultneh, Y. & Butcher, R. J. (2017). *Acta Cryst.* E**73**, 1708–1711. 10.1107/S2056989017014694 PMC5683496 29152356

[bb16] Okeke, U. C., Gultneh, Y., Jasinski, J. P. & Butcher, R. J. (2019). *Inorg. Chem. Commun.* **102**, 45–50.

[bb17] Okeke, U. C., Gultneh, Y., Otchere, R. & Butcher, R. J. (2018). *Inorg. Chem. Commun.* **97**, 1–6.

[bb18] Rigaku OD (2024). *CrysAlis PRO*. Rigaku Oxford Diffraction, Yarnton, England.

[bb19] Romary, J. K., Zachariasen, R. D., Barger, J. D. & Schiesser, H. (1968). *J. Chem. Soc. C* pp. 2884–2887.

[bb20] Sheldrick, G. M. (2008). *Acta Cryst.* A**64**, 112–122. 10.1107/S0108767307043930 18156677

[bb21] Sheldrick, G. M. (2015*a*). *Acta Cryst.* A**71**, 3–8.

[bb22] Sheldrick, G. M. (2015*b*). *Acta Cryst.* C**71**, 3–8.

[bb23] Uhlig, E., Borek, B. & Glänzer, H. (1966). *Z. Anorg. Allg. Chem.* **348**, 189–200.

